# Sodium Alginate/MXene-Based Flexible Humidity Sensors with High-Humidity Durability and Application Potentials in Breath Monitoring and Non-Contact Human–Machine Interfaces

**DOI:** 10.3390/nano14211694

**Published:** 2024-10-23

**Authors:** Huizhen Chen, Xiaodong Huang, Yikai Yang, Yang Li

**Affiliations:** MOE Key Laboratory of Macromolecular Synthesis and Functionalization, Department of Polymer Science and Engineering, Zhejiang University, Hangzhou 310058, China; 12229001@zju.edu.cn (H.C.); 22329021@zju.edu.cn (X.H.); 3190103753@zju.edu.cn (Y.Y.)

**Keywords:** flexible humidity sensor, sodium alginate, MXene, high-humidity durability, breath monitoring, non-contact human–machine interface

## Abstract

Flexible humidity sensors (FHSs) with fast response times and durability to high-humidity environments are highly desirable for practical applications. Herein, an FHS based on crosslinked sodium alginate (SA) and MXene was fabricated, which exhibited high sensitivity (impedance varied from 10^7^ to 10^5^ Ω between 10% and 90% RH), good selectivity, prompt response times (response/recover time of 4 s/11 s), high sensing linearity (R^2^ = 0.992) on a semi-logarithmic scale, relatively small hysteresis (~5% RH), good repeatability, and good resistance to highly humid environments (negligible changes in sensing properties after being placed in 98% RH over 24 h). It is proposed that the formation of the crosslinking structure of SA and the introduction of MXene with good conductivity and a high specific surface area contributed to the high performance of the composite FHS. Moreover, the FHS could promptly differentiate the respiration status, recognize speech, and measure fingertip movement, indicating potential in breath monitoring and non-contact human–machine interactions. This work provides guidance for developing advanced flexible sensors with a wide application scope in wearable electronics.

## 1. Introduction

In recent years, flexible humidity sensors (FHSs) have received considerable attention in respiratory monitoring [1,2,3,4,5], speech recognition [6,7,8], and non-contact human–machine interfaces (HMIs) [9,10,11,12,13,14], demonstrating great potential for use in wearable electronics. In practical applications, the humidity sensors usually have to work in high-humidity environments. For instance, when used for the real-time monitoring of breath, the humidity sensors inevitably contact exhaled gas with high humidity (relative humidity (RH) of ~90%) and thus should be resistant to humid environments. Moreover, the humidity sensors should possess fast response times in order to keep up with the high frequency of breath in certain scenarios (for example, fast breath after exercises). Furthermore, non-contact HMIs also rely on the ability of the FHS to promptly respond to humidity changes in the micro-environment induced by fingertip movement [15]. In consequence, it is necessary to develop FHSs with good durability to humid environment and fast response to satisfy the requirements for applications.

It is well known that the sensing performance of an FHS is highly dependent on the humidity sensing materials. MXene is a new class of two-dimensional transition metal carbides, nitrides, or carbon nitrides with advantages of metal conductivity and mechanical robustness, acting as an attractive alternative to conventional metal- and carbon-based materials [16]. Its unique accordion-like structure endows the material with a high specific surface area to facilitate the adsorption and desorption of water molecules. In addition, its surface is deposited with -OH, -F, -O, and other terminal functional groups. Therefore, MXene shows great application potential in electromagnetic shielding [17], gas sensors [18], electrochemical sensing [19], catalysis [20], etc. Due to the high electrical conductivity, large specific surface area, and abundant hydrophilic functional groups, MXene is a research hotspot in humidity sensing. However, the response sensitivity of MXene alone is limited, which can be effectively improved by compounding it with polyelectrolytes [21].

Polyelectrolytes are promising humidity sensing materials with features of high sensitivity, easy preparation, etc. However, they generally show slow response times/recovery speeds and poor stability in high levels of humidity due to their low specific surface area and water-solubility in high-humidity environment. To tackle these problems, blending with nanostructured materials to improve the specific surface area of sensitive films and introduce crosslinked structures offer effective solutions [22,23,24,25].

Sodium alginate (SA) is a biopolymer derived from seaweeds, consisting of (1–4)-linked β-D-mannuronic acid (M) and α-L-guluronic acid (vii) residue. It has been used in many fields and has proved to be a promising low-cost, low-density functional material [26]. The use of renewable SA as a matrix also exhibits eco-friendliness and biodegradability compared to traditional petroleum-based polymers. SA is rich in hydroxyl groups and can be used as a humidity sensing material. However, the poor electrical conductivity of SA leads to high impedance in low/medium humidity when the material is used as a moisture-sensitive material and low sensitivity to low and medium humidity. Combining the advantages of MXene and SA is expected to yield humidity sensors with high sensitivity. Zhao et al. [27] fabricated a composite film of L-citrulline-modified MXene with SA with humidity responsivity, in which the electrostatic interactions between positively charged modified MXene and negatively charged SA lead to a crosslinking structure. However, the modification of MXene is relatively cumbersome with long preparation times. Moreover, the examination of the humidity responsivity, which is realized by the measurement of the weights, thicknesses, currents, or resistances of the composite film, represents quite a slow process, lasting for several hours, and is thus unbeneficial for practical applications. Dong and coworkers [28] prepared multifunctional SA@MXene-based coaxial fibers via wet spinning to realize humidity sensing. But the humidity sensitivity of this coaxial fiber (relative resistance change of ~2% from 7%RH to 84%RH) is much smaller than the polyelectrolyte-based humidity sensors (typical impedance change of several orders of magnitude when changed from dry to humid environment), which might limit the actual use.

Herein, an FHS was fabricated based on a nanocomposite of crosslinked SA and MXene. Specifically, polyimide (PI) film was employed as the flexible substrate of the sensor, on which interdigitated electrodes (IDEs) were constructed by using the method of laser direct writing (LDW) to convert PI to conductive graphene. The composite sensing material of SA/MXene loaded on the surface of the PI film was further treated with CaCl_2_ to introduce a cross-linking structure to improve the durability to high humidity. Owing to the large specific surface area of MXene and good water-absorbing ability of SA, the as-prepared FHS exhibited fast response times, good sensitivity, high selectivity, and good sensing repeatability. In particular, the crosslinked network was introduced and the FHS showed little change in the sensing performance even after being placed in a humid environment for up to 24 h, revealing high resistance to high humidity. Moreover, it could successfully realize respiratory monitoring, speech recognition, and the detection of slight movements of fingertips in a non-contact way. The work offered an effective solution for the development of advanced flexible nanosensors with fast response times and good durability towards humid environments with a wide application scope.

## 2. Experimental

### 2.1. Materials and Reagents

Ti_3_AlC_2_ (400 mesh, >99.5%) was provided by Shandong Xiyan New Material Science and Technology Co., Ltd. (Tai’an, China) Hydrogen fluoride (HF, AR), sodium alginate (SA, CP), sodium hydroxide(NaOH, AR), lithium chloride(LiCl, 99%), magnesium chloride hexahydrate (MgCl_2_·6H_2_O, AR), sodium bromide (NaBr, AR), sodium chloride (NaCl, AR) and potassium sulfate(K_2_SO_4_, AR) were purchased form Sinopharm Chemical Reagent Co., Ltd. (Shanghai, China). Calcium chloride (CaCl_2_, AR) was obtained from Quzhou Juhua Reagent Co., Ltd. (Quzhou, China). Polyimide (PI, 0.2 mm) was supplied by Shenzhen Yingshida Plastic Materials Co., Ltd. (Shenzhen, China).

### 2.2. Preparation of Crosslinked SA (c-SA)/MXene FHS

**Figure 1:**
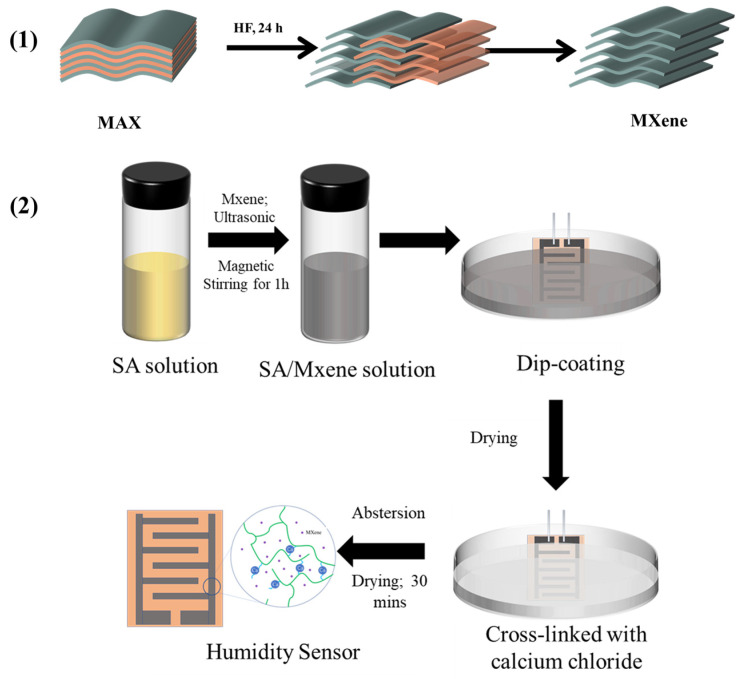
Schematic illustration for the preparation of (1) MXene and (2) c-SA/MXene FHS.

The preparation process of the composite FHS is shown in Figure 1. First, MXene was prepared by etching Ti_3_AlC_2_ (MAX phase) with HF according to a method described in previous research [29,30]. Typically, 1 g Ti_3_AlC_2_ powder and 15 mL HF were added into a 50 mL Teflon reactor and magnetically stirred for 24 h. The resultant was centrifuged at 5000 r/min for 5 min, and the isolated solid product was redispersed in water. The operation was repeated several times until the pH of the supernatant was ~6. The isolated solid product was freeze-dried for 4 h using a lyophilizer (FD-1A-50, Beijing Boyikang Experimental Instrument Co., Ltd., Beijing, China) to obtain MXene powder. HF is a corrosive and toxic reagent. When using HF, it is necessary to wear nitrile gloves and a gas mask and to use plastic containers to store HF. After the reaction, HF needs to be carefully neutralized before being collected as an inorganic waste.

**Figure 2:**
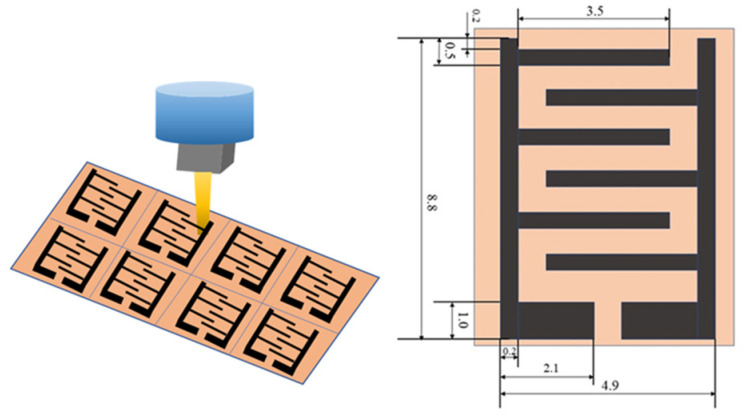
Preparation of IDEs.

PI film was hydrophilically modified by soaking in NaOH solution (1 mol/L) for 30 min, washed with deionized water thoroughly, and dried at 80 °C for 30 min. IDEs were fabricated on the modified PI film by a CO_2_ laser engraver to convert PI into graphene (scanning speed: 100 mm/s, power: 9.6 W, line spacing: 1 mm, Tianjin Jiayin Nanotechnology Co., Ltd., Tianjin, China). The scheme of IDEs is shown in Figure 2.

As shown in Figure 1(2), various amounts of MXene were added into aqueous solution of SA (4 mg/mL) and magnetically stirred for 1 h to prepare a uniform dispersion of SA/MXene. The PI films with IDEs were dip-coated in the dispersion of SA/MXene for 1 min and then dried at 50 °C for 30 min. Dip-coating is a popular method to fabricate thin-film devices including sensors, with advantages of simplicity, high efficiency, and good reproducibility. The as-prepared sample was named as SA/MXeneα, where α is the concentration of MXene in the dispersion in mg/mL. Subsequently, the sample was immersed in CaCl_2_ solution (5 wt%) for the crosslinking of SA, washed with deionized water, and dried at 50 °C for 30 min. The resulting FHS was named c-SA/MXeneα-β, where β is the crosslinking time in min.

### 2.3. Characterizations

Morphologies of Ti_3_AlC_2_, MXene, FHSs of SA/MXeneα, and c-SA/MXeneα-β were analyzed by field emission scanning election microscopy (FE-SEM, Hitachi SU-8010, Tokyo, Japan). Fourier transform infrared (FTIR) spectra (KBr pellets) and attenuated total reflection infrared Fourier transform infrared (ATR-FTIR) spectra were obtained on a Nicolet 6700 FTIR spectrophotometer (Themo Fisher scientific LLC, Waltham, MA, USA). Raman spectrum was collected on a Raman spectrophotometer (INVIA-REFLEX, Renishaw plc, London, UK) with a He-Ne laser (λ = 532 nm).

### 2.4. Measurements of Humidity Sensing Properties

**Figure 3:**
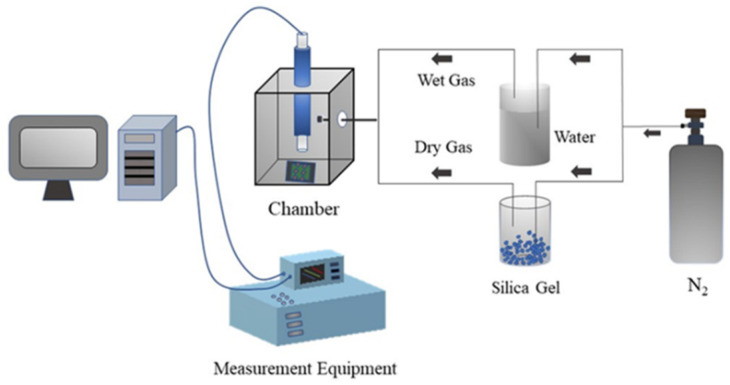
Schematic diagram of humidity testing device.

The real-time humidity response of the FHS was measured with a home-made device in a chamber where the relative humidity (RH) was controlled by adjusting the mixed ratio of dry and wet gasses and was calibrated with a commercial hygrometer (Rotronic, Hygroclip HC2-S3, Bassersdorf,, Switzerland) at room temperature (~25 °C). The test voltage was AC 1 V with a frequency of 1 kHz. A diagram of the humidity testing device is shown in Figure 3.

The response/recovery time (t_90%_) of the sensor is defined as the time required for the impedance of the sensor to reach 90% of the total change.

To study the stability of the FHS in a humid environment, the sensor was placed under 98% RH for 9 h, and its impedance was recorded online using an intelligent LCR meter (4092C, Shenzhen Yisheng Victory Technology Co., Ltd., Shenzhen, China). Furthermore, the FHS was put under 98% RH for 24 h, and then the sensing performance of the FHS before and after treatment in the high-humidity environment was tested. The ambient humidity was 70% RH (±1% RH), and the temperature was 22 °C (±0.5 °C). The test voltage was AC 1 V and the frequency was 1 kHz.

To test the effect of organic vapors on the impedance response of the FHS, the impedance of the c-SA/MXene1-30 FHS before and after exposure to organic vapors (ethanol, ether, methanol, acetone, and n-hexane) was measured at 74% RH and 27 °C. Specifically, the calculated amount of the liquids of the organic solvents was injected into a vessel fitted with a small electric fan for the volatilization of the liquid to obtain organic vapors of 500 ppm. The sensitivity (*S*) of the FHS to different organic vapors is defined as follows:

(1) $$S\left(\%\right)=\frac{Z-Z_{0}}{Z_{0}}\times 100$$

where *Z* and *Z*_0_ are the impedance before and after exposure to organic vapors for 5 min, respectively.

The repeatability of the c-SA/MXene1–30 FHS was examined by recording its impedance in real time when the sensor was switched between high-humidity (98% RH) and dry (11% RH) environments at an interval of 30 s for 120 cycles at room temperature (~25 °C).

Nyquist plots of the sensor under different humidity levels (11, 33, 59, 75, and 98% RH) were recorded on a ZL5 intelligent LCR meter (Shanghai Haoshun technology Co., Ltd., Shanghai, China) at room temperature. Various stable humidity environments were provided by using saturated salt solutions (LiCl: 11% RH; MgSO_4_: 33% RH; NaBr: 59% RH; NaCl: 75% RH; K_2_SO_4_: 98% RH).

### 2.5. Application of c-SA/MXene1-30 FHS

(1) Real-time respiratory monitoring: The FHS was placed close to the mouth or under the nose of a volunteer, and the real-time impedance of the sensor during the respiration in different modes was recorded using a ZL5 intelligent LCR meter. The ambient humidity was 51% RH (±1% RH), and the temperature was 25 °C (±0.5 °C).

(2) Speech recognition: The FHS was displayed in front of the lip of the volunteer, and the real-time impedance of the sensor was recorded with a ZL5 intelligent LCR meter. The volunteer spoke different words, such as “pen”, “paper”, and “potato”, and the corresponding signal was recorded. The ambient humidity was 75% RH (±1% RH), and the temperature was 25 °C (±0.5 °C).

(3) Non-contact detection of fingertip movement: The impedance change in the FHS was recorded online with a ZL5 intelligent LCR meter when the fingertip of the volunteer cyclically moved between a sufficiently far place (distance > 40 cm) and a place close to the sensor (distances: 8 mm, 3 mm, and 1 mm). The ambient humidity was 75% RH (±1% RH), and the temperature was 25 °C (±0.5 °C).

## 3. Results and Discussion

### 3.1. Characterization of FHS

**Figure 4:**
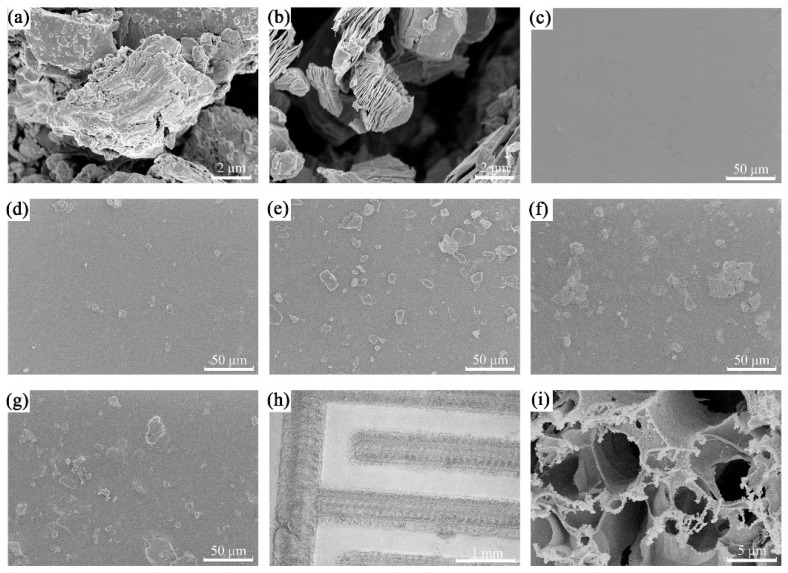
SEM images of (**a**) MAX phase (Ti_3_AlC_2_); (**b**) MXene; (**c**) SA; (**d**) SA/MXene0.5; (**e**) SA/MXene1; (**f**) SA/MXene2; (**g**) c-SA/MXene1-30; IDEs at (**h**) low magnification and (**i**) high magnification.

In this work, we prepared MXene by etching with HF, as shown in Figure 1, and the SEM images of the MAX phase and MXene are illustrated in Figure 4a,b. It is seen that the MAX phase comprises uneven and tightly packed blocks. In comparison, as-prepared MXene shows an accordion-like structure, which is in agreement with the literature report [30]. The larger specific surface area of MXene than the bulk SA film can provide more active sites and can benefit the adsorption and desorption of water molecules. Figure 4c–g present the morphology of the FHS with different concentrations of MXene. SA alone exhibits a smooth surface, while MXene debris is clearly observed on the surface of the composite of SA/MXene. With the increment of the concentration of MXene, the composite exhibits a rougher surface with a higher density of MXene debris. Furthermore, the composite film shows little change in the morphology after the crosslinking of SA with CaCl_2,_ as evidenced by the comparison of Figure 4f,g.

**Figure 5:**
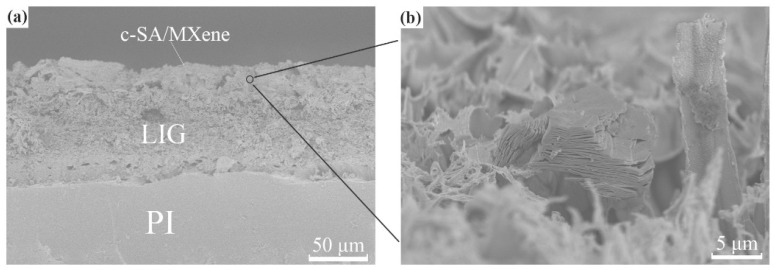
SEM images of (**a**) cross section of c-SA/MXene1-30 FHS and (**b**) c-SA/MXene layer on IDEs at a high magnification scale.

Figure 4h,i present the SEM images of IDEs constructed on PI film. The boundary of the area treated with LDW and without LDW treatment is clear, indicating the high resolution of the patterned structure induced by LDW treatment. At high magnification, IDEs reveal rugged and porous surface, which is attributed to the conversion of PI to the conductive graphene by laser treatment and is in agreement with literature reports [31,32,33,34]. The SEM micrograph of the cross-sectional view of the fabricated device is presented in Figure 5a, and the three parts of the compact PI, laser-induced graphene, and superimposed MXene/SA are identified. A magnified view of the MXene/SA layer in Figure 5b illustrates the accordion-shaped MXene.

**Figure 6:**
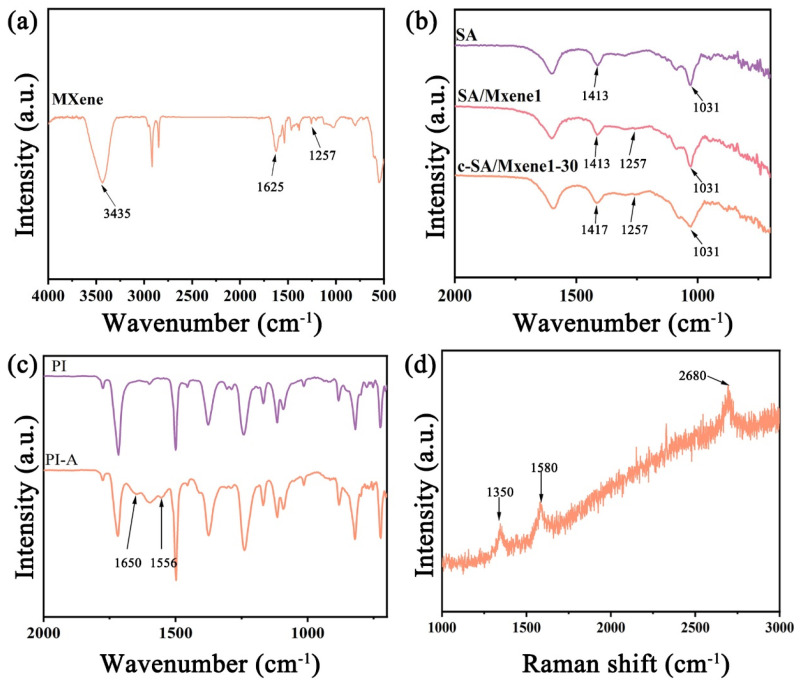
(**a**) FTIR spectrum of MXene; ATR-FTIR spectra of (**b**) SA, SA/MXene1, and c-SA/MXene1-30; (**c**) PI and PI-A; (**d**) Raman spectrum of IDEs in PI.

The chemical structure of the samples has been examined by the analysis of FTIR spectroscopy. In the FTIR spectrum of MXene (Figure 6a), the absorption peaks at 3435 cm^−1^ and 1625 cm^−1^ are ascribed to hydrogen-bonded -OH or water molecules coordinated on the surface of MXene. Furthermore, the characteristic peak at 1257 cm^−1^ is attributed to the stretching vibration of C-F [35]. The FTIR characterization indicates the existence of functional groups, like -OH and -F, attached to the surface of MXene.

Figure 6b shows the ATR-FTIR spectra of SA, SA/MXene1, and c-SA/MXene1-30. In the spectrum of SA, the absorption peak at 1031 cm^−1^ represents the symmetric stretching vibration of C-O on C-O-C [36]. The characteristic peak 1413 cm^−1^ is attributed to the symmetric stretching of –COO– on the polymer chain [37]. In the spectrum of MXene, the peak at 1257 cm^−1^ is ascribed to C-F in MXene [35]. Both characteristic peaks of MXene and SA are clearly identified in the spectrum of SA/MXene1. In comparison, in the spectrum of c-SA/MXene1-30, the absorption peak corresponding to the symmetric stretching of –COO– on SA is shifted from 1413 cm^−1^ to 1417 cm^−1^, which is attributed to the crosslinked structure between CaCl_2_ and SA [37]. The above results indicate the successful preparation of SA/MXene and c-SA/MXene.

The ATR-FTIR spectra of PI and PI-A are shown in Figure 6c. Compared with PI, in the ATR-FTIR spectrum of PI-A, new absorption peaks appear at 1556 cm^−1^ and 1650 cm^−1^, which are assigned to the N-H bending vibration peaks and C=O stretching vibration peaks, respectively. The observation of such new functional groups proves the hydrolysis of the imide group of PI by the treatment with NaOH [38].

Figure 6d shows the Raman spectrum of the IDEs formed by laser treatment in the PI substrate. Three prominent peaks in the spectrum are identified at 1350 cm^−1^, 1580 cm^−1^, and 2680 cm^−1^, corresponding to the D band, G band, and 2D band of graphene, respectively [39]. The D band is related to the conversion of sp^2^-hybridized carbon to sp^3^-hybridized carbon, while the G band is related to the vibration of sp^2^-hybridized carbon [40]. Obviously, the Raman characterization reveals that the LDW-induced IDEs are composed of conductive graphene.

### 3.2. Humidity Sensing Properties of FHS

**Figure 7:**
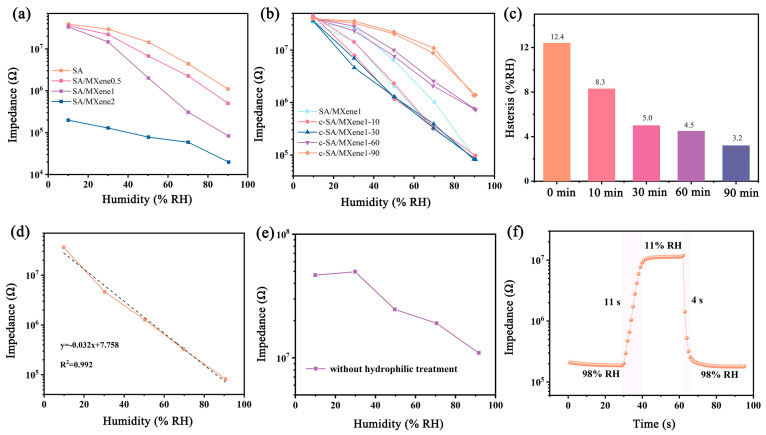
Humidity sensitive curves of FHS prepared with (**a**) different concentration of MXene; (**b**) differ-ent crosslinking time; (**c**) hysteresis of FHS with different crosslinking time; (**d**) humidity sensitive curve of c-SA/MXene1-30 FHS; (**e**) humidity sensitive curve of c-SA/MXene1-30 FHS based on PI substrate without hydrophilic treatment; (**f**) response and recovery time of c-SA/MXene1-30 FHS.

Figure 7a presents the humidity sensing curves of the SA/MXene FHS with different concentrations of MXene. The impedance of the FHS based on SA varies from ~10^6^ Ω to 10^7^ Ω within the range of 90% RH to 10% RH, showing relatively low sensitivity and high impedance even under humid environments. It is well known that SA is a kind of natural polyelectrolyte with a large number of hydrophilic functional groups. With the increase in RH, more water molecules are absorbed in SA film, which are decomposed to produce protons and also promote the dissociation of Na^+^ in SA to improve ion conductivity, leading to the decrement of impedance. However, the sensitivity of SA is limited, which can be further improved by compositing with nanomaterials with large specific areas and high conductivity. Herein, MXene was introduced into the sensitive film, and the resulting sensing curves are shown in Figure 7a. The FHS based on the composite of SA/MXene exhibited much lower impedance than that based on SA alone at every RH investigated, which is attributed to the excellent conductivity of MXene. Moreover, the composite sensor revealed much higher sensitivity than the sensor of SA. Mxene possesses a higher specific surface area than bulk SA film and has abundant hydrophilic groups (such as -OH), which could provide sufficient active sites for the adsorption of water molecules and therefore improve the sensitivity of the composite film. Specifically, when the concentration of MXene in the composite solution reaches 2 mg/mL, the impedance of the corresponding sensor decreased dramatically over the whole tested humidity range. It is proposed that the conducting path was well established in the composite film at such a high concentration of highly conductive MXene, leading to the greatly improved conductivity of the composite sensor. Therefore, the variation of adsorbed water molecules with the change in RH did not result in great changes in the conductivity, and the sensor demonstrated lower sensitivity. In the work, 1 mg/mL of MXene was selected as the optimal formula for the fabrication of the composite FHS.

Figure 7b shows the influence of crosslinking time on the humidity sensing properties of the c-SA/MXene FHS. The FHS of SA/MXene exhibited a quite large hysteresis of ~12.5% RH. In comparison, the FHS based on c-SA/MXene obtained by immersion in CaCl_2_ solution showed decreased hysteresis. Specifically, extending the immersion time of the FHS in CaCl_2_ solution leads to smaller hysteresis and higher sensitivity. It has been reported that SA could react with Ca^2+^ to form a stable intermolecular cross-linking network structure [41]. Apparently, such a crosslinked structure is helpful in hindering the accumulation of water molecules and avoiding the formation of a water layer in the sensing film, resulting in smaller hysteresis of the FHS. When the cross-linking time reaches 60 min, the sensor exhibited a relatively small hysteresis of ~5.0% RH. Further increasing the immersion time could lead to even smaller hysteresis, but the sensitivity of the FHS is decreased too. An immersion time of 30 min is thus chosen as the optimal formula by considering the sensitivity and hysteresis. The c-SA/MXene1-30 FHS exhibited an impedance change of two orders of magnitude and good sensing linearity (R^2^ = 0.992, Figure 7d) on a semi-logarithmic scale over a wide range of 11–98% RH. Moreover, it revealed short response/recovery times (4 ± 0.9 s/11 ± 1.1 s, Figure 7f), which might be ascribed to the high specific surface area of MXene and the accelerated desorption of water molecules due to the formation of the crosslinked structure.

Additionally, the humidity sensing curve of the FHS based on c-SA/MXene1-30 with PI without hydrophilic treatment as the substrate is presented in Figure 7e to explore the effect of surface modification on the humidity response of the FHS. The sensor with unmodified hydrophobic PI as the substrate showed very high impedance of over 10^7^ Ω in the whole tested humidity range (10–90% RH), with slight variation in the impedance. It is proposed that the incompatibility between a hydrophobic substrate and hydrophilic sensitive materials hindered the deposition of the sensitive film on the flexible substrate, leading to poor sensitivity of the resulting FHS. Apparently, the results revealed the importance of the NaOH treatment of the PI film for the fabrication of the high-performance composite FHS.

**Figure 8:**
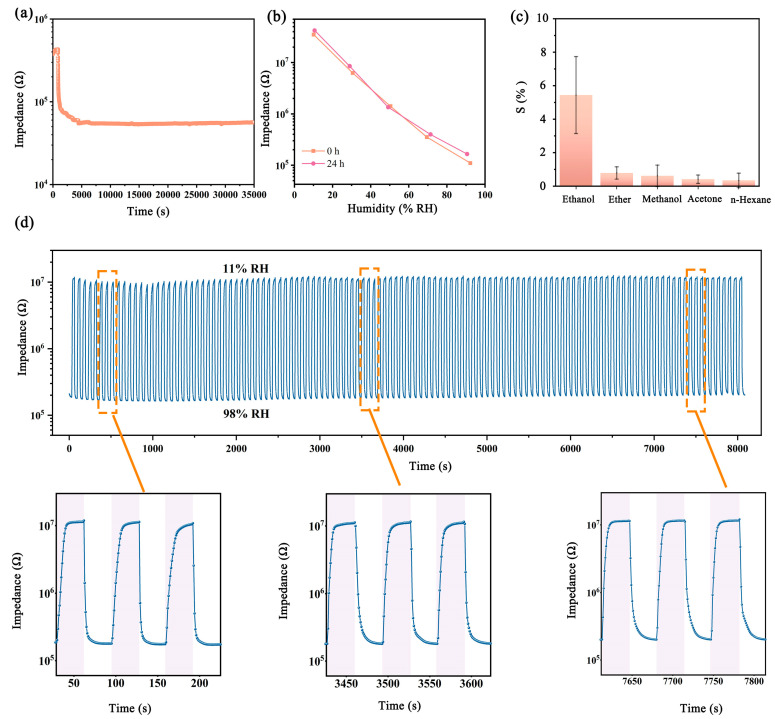
(**a**) Real-time response of the c-SA/MXene1-30 FHS placed in 98% RH for over 9 h; (**b**) humidity response of the c-SA/MXene1-30 FHS when the FHS was placed in 98% RH of 0 h and 24 h; (**c**) response of the c-SA/MXene1-30 FHS when exposed to 500 ppm vapors of different analytes: ethanol, ether, methanol, acetone, n-hexane; (**d**) response of the c-SA/MXene1-30 FHS during cyclic switching between high- and low-humidity environments.

As discussed before, the stability under a humid environment is crucial for the practical applications of the FHS. Herein, the c-SA/MXene1-30 FHS was placed in 98% RH for over 9 h, and the real-time impedance response is illustrated in Figure 8a. Clearly, the FHS did not show any degradation in sensing performance during the storage in the highly humid environment. Furthermore, the FHS was placed in a bottle containing water at 40 °C for 24 h, and its humidity responses before and after the exposure to the humid environment changed little, as shown in Figure 8b. The results clearly demonstrated that the FHS exhibited good durability towards humid environments, which might relate to the formation of the crosslinking structure of SA in the composite film. Additionally, the responses of the c-SA/MXene1-30 FHS to organic gasses (methanol, ethanol, ether, acetone, and n-hexane) are presented in Figure 8c. The FHS exhibited quite small responses towards all of the tested organic gasses, demonstrating excellent selectivity of the c-SA/MXene1-30 FHS. Figure 8d displays the real-time responses of the c-SA/MXene1-30 FHS during quick switching between high- and low-humidity environments for 120 cycles at an interval time of 30 s. Clearly, the early, middle, and late stages of the sensing curves show almost the same impedance values at high- and low-humidity levels and unchanged shapes, suggesting the good sensing repeatability and stability of the FHS.

**Figure 9:**
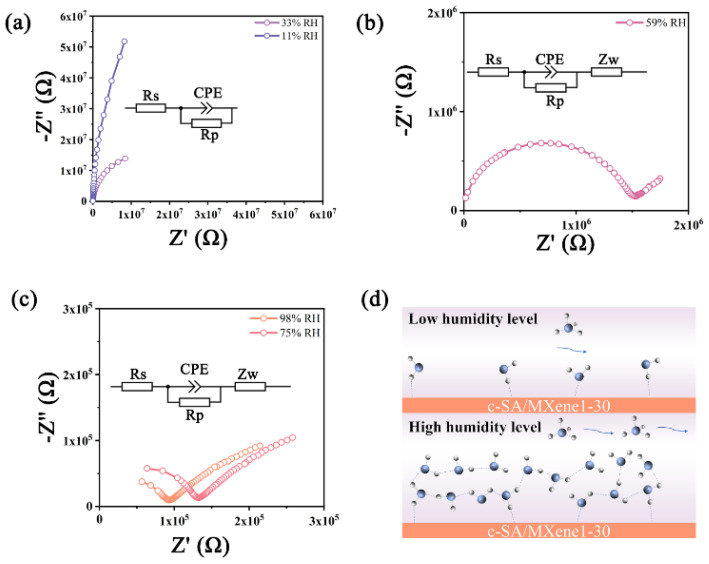
Nyquist plots of the c-SA/MXene1-30 FHS: (**a**) under 11 and 33% RH, (**b**) under 59% RH, (**c**) under 75 and 98% RH; (**d**) diagram of proposed sensing mechanism.

Nyquist plots of the c-SA/MXene1-30 FHS under various humidity environments were measured to explore the humidity sensing mechanism. As shown in Figure 9a, when the humidity is below 33% RH, the Nyquist plot of the sensor is composed of a semicircle. In comparison, the Nyquist plots are composed of a semicircle at high frequencies and a straight line at low frequencies under 59–98% RH. The semicircle represents the membrane impedance of the sensor, while the straight line is ascribed to the Warburg impedance of the diffusion process [42]. Specifically, at low humidity levels (11% RH, 33% RH), the corresponding equivalent circuit was described by a parallel resistor and capacitor circuit. In this situation, only a few water molecules could form hydrogen bonds with hydrophilic groups on MXene and -OH on the SA main chain, and were adsorbed on the surface of the sensitive membrane to form a discontinuous water layer. At this point, the sensor relies on the hopping of H^+^ to achieve charge transfer [43]; thus, the impedance is relatively high. At 59% RH, the water molecules form a continuous water layer on the surface of the sensitive film due to the increase in humidity [44], prompting the ionization of Na^+^ in SA, resulting in a decrease in the impedance of the sensor. Due to the diffusion of ions at the interface, the radius of the semicircle decreases and a straight line representing the Warburg impedance (Zw) appears in the low-frequency region, which is equivalent to a circuit with Zw connected in series to the parallel circuit described above. As the humidity increases, the mobility of the conducting ions (H^+^, H_3_O^+^, Na^+^) also increases, resulting in a drop of impedance and a decrease in the radius of the semicircle.

### 3.3. Applications of FHS

**Figure 10:**
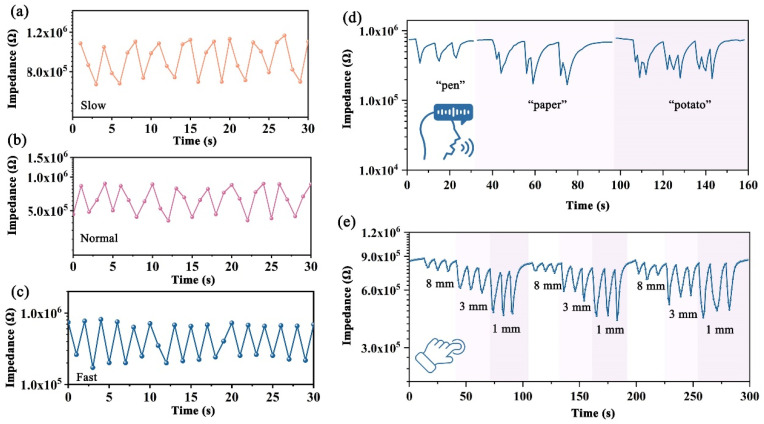
Application of c-SA/MXene1-30 FHS: respiration monitoring under different states: (**a**) slow speed; (**b**) normal speed; (**c**) fast speed; (**d**) speech recognition; (**e**) real-time impedance response of distance change between sensor and fingertips.

The exhaled air of a human is highly humid and could change the humidity in the surrounding microenvironment [45]. Therefore, the detection of breath-induced humidity changes could be utilized to reflect the respiration status for non-invasive and non-contact early diagnosis [46,47]. Herein, the c-SA/MXene1-30 FHS was assembled in a mask and worn by a volunteer to verify its potential for respiratory monitoring. The real-time responses of the FHS towards the respiration of the volunteer at different breathing modes are shown in Figure 10a–c. It is seen that the response curves exhibit distinct amplitude and frequency towards three different breathing modes. Apparently, the response curves exhibited the largest amplitude and the lowest frequency towards the slow breathing mode, while the sensing curves with the smallest amplitude and the highest frequency indicated the fast breathing mode. It is proposed that during the breathing process, exhaled gas carries a large number of water molecules, which increases the humidity of the air around the mouth and leads to a decrease in the impedance of the sensor. By contrast, the water vapor content is lowered during inhalation and the humidity around the mouth decreases, resulting in higher impedance for the sensor. In a slow breathing rate, the FHS could adsorb/desorb water molecules more efficiently and thus could exhibit larger impedance changes and greater amplitude in the sensing curve. Obviously, our FHS with fast response times could differentiate well between the respiratory patterns, revealing potential in non-contact respiratory motoring.

Similarly, the exhaled airflow during speaking leads to the vibration of the vocal cords to produce sound, which is then modulated into different syllables through the mouth and nose to form specific words. Apparently, the process of speaking is accompanied by humidity changes in the air around the mouth. Therefore, the detection of the humidity change in the microenvironment could be utilized to distinguish between the different syllables. In this work, a volunteer said different words (monosyllabic (pen), disyllabic (pencil), and trisyllabic (tomato)), and the real-time responses of the c-SA/MXene1-30 FHS assembled around the mouth of the speaker was recorded (Figure 10d). It is clear that the FHS exhibited characteristic and repeatable sensing curves towards various syllables, indicating the potential of speech recognition by the c-SA/MXene1-30 FHS.

There have been a number of reports on the application of humidity sensors in non-contact HMIs [48,49,50]. Herein, the real-time impedance response of the c-SA/MXene1-30 FHS was recorded when the fingertip of a volunteer approached the sensor at different distances of 1 mm, 3 mm, and 8 mm, and the results are displayed in Figure 10e. It was found that the FHS exhibited sensitive, reversible, and repeatable responses towards the change in the distance between the sensor and fingertip, proving its capability in detecting the delicate movement of fingertips in a non-contact way. Obviously, the results indicate that the c-SA/MXene1-30 FHS shows potential for non-contact HMIs, which can be used to reduce the risk of virus transmissions, such as COVID-19, during large-scale infectious disease outbreaks.

## 4. Conclusions

An FHS was facilely fabricated by depositing the nanocomposite of crosslinked SA and MXene on a NaOH-modified PI substrate. The composite FHS exhibited fast response times and good durability towards high-humidity environments due to the introduction of 2D nanomaterials of MXene with high specific surface areas and good conductivity and the formation of stable crosslinking structures between SA and Ca^2+^. Moreover, the as-prepared FHS exhibited high sensitivity (impedance change of two orders of magnitude between 10% and 90% RH), good selectivity, and sensing repeatability. The high-performance FHS demonstrated great potential for the monitoring of respiration, speech recognition, and non-contact HMIs by detecting the delicate movements of fingertips. This work provides a feasible approach for improving the sensing performance of flexible nanosensors by simply modifying the structure and composition of the sensing film to satisfy the requirements for practical applications in wearable electronics.

## Data Availability

Data are contained within the article.
